# Colil: a database and search service for citation contexts in the life sciences domain

**DOI:** 10.1186/s13326-015-0037-x

**Published:** 2015-10-19

**Authors:** Toyofumi Fujiwara, Yasunori Yamamoto

**Affiliations:** INTEC Inc, 1-3-3 Shinsuna, Koto-ku, Tokyo, 136-8637 Japan; Database Center for Life Science, Research Organization of Information and Systems, 178-4-4 Wakashiba, Kashiwa-shi, Chiba 277-0871 Japan

**Keywords:** Life sciences paper, Citation, Citation context, Co-citation, PMC Open Access Subset, RDF, SPARQL

## Abstract

**Background:**

To promote research activities in a particular research area, it is important to efficiently identify current research trends, advances, and issues in that area. Although review papers in the research area can suffice for this purpose in general, researchers are not necessarily able to obtain these papers from research aspects of their interests at the time they are required. Therefore, the utilization of the citation contexts of papers in a research area has been considered as another approach. However, there are few search services to retrieve citation contexts in the life sciences domain; furthermore, efficiently obtaining citation contexts is becoming difficult due to the large volume and rapid growth of life sciences papers.

**Results:**

Here, we introduce the Colil (Comments on Literature in Literature) database to store citation contexts in the life sciences domain. By using the Resource Description Framework (RDF) and a newly compiled vocabulary, we built the Colil database and made it available through the SPARQL endpoint. In addition, we developed a web-based search service called Colil that searches for a cited paper in the Colil database and then returns a list of citation contexts for it along with papers relevant to it based on co-citations. The citation contexts in the Colil database were extracted from full-text papers of the PubMed Central Open Access Subset (PMC-OAS), which includes 545,147 papers indexed in PubMed. These papers are distributed across 3,171 journals and cite 5,136,741 unique papers that correspond to approximately 25 % of total PubMed entries.

**Conclusions:**

By utilizing Colil, researchers can easily refer to a set of citation contexts and relevant papers based on co-citations for a target paper. Colil helps researchers to comprehend life sciences papers in a research area more efficiently and makes their biological research more efficient.

**Electronic supplementary material:**

The online version of this article (doi:10.1186/s13326-015-0037-x) contains supplementary material, which is available to authorized users.

## Background

The ability to efficiently identify current research trends, advances, and issues in a research area is highly important to researchers to promote their research activities. For example, in cases of international collaborative research, researchers often need to read relevant papers and summarize the current knowledge about the research area beyond their own research fields [1]. Although review papers in the research area can suffice for this purpose in general, researchers are not necessarily able to obtain these papers at the right time from viewpoints of their interests. In addition, these papers reflect only previously published papers at the time of writing a review paper and viewpoints of its authors. To complement this issue, the utilization of the citation contexts for papers in a research area has been considered as another approach [2]. However, there are few search services to retrieve citation contexts in the life sciences domain, and to efficiently obtain the citation contexts for a target paper is becoming difficult due to the large volume and rapid growth of life sciences papers. Here, we introduce the Colil (Comments on Literature in Literature) database and a web-based search service called Colil for citation contexts in the life sciences domain.

In life sciences papers, citations are widely used and typically consist of two parts: a) a list of references found at the end of the citing paper that provides full bibliographic information for each source; and b) reference markers located in the text that are linked to the references [3]. The text surrounding a reference marker is defined as the citation context [4]; it contains information about the cited paper such as the important contributions of it, criticism against it, comparison of the work in it to the author’s work, or use of the method described in it [5]. In previous studies, Bradshaw [6] showed that citation contexts provide many perspectives on a paper. Qazvinian and Radev [2] and Mei and Zhai [7] argued that citation contexts are useful for creating a summary of the important aspects of a paper. Furthermore, Elkiss et al. [8] and Divoli et al. [9] examined the relationships between the abstract and the citation contexts of a given life sciences paper, and their experiments showed that citation contexts tend to have additional and focused information that is not present in the abstract. These results indicate that citation contexts play an important role in representing the semantic content of life sciences papers.

To make better use of citation data, we built the Colil database as Linked Open Data (LOD) by using the Resource Description Framework (RDF). RDF is a promising technology for describing, publishing, and linking life sciences data on the Web [10]. In addition, LOD are linked to other related resources that use RDF and is released under an open license [11]. These technologies have the potential to facilitate data integration and provide the semantics to perform rich queries using the SPARQL query language [10]. To build the Colil database as LOD, we use a newly compiled vocabulary called Comments on Literature in Literature Ontology (COLILO) in addition to standard existing vocabularies such as Bibliographic Ontology (BIBO) [12], Dublin Core Metadata Initiative Metadata Terms (DCTERMS) [13], and Document Components Ontology (DoCO) [14]. We linked resources in the Colil database to their corresponding external ones such as Biotea [15], PubMed Central (PMC) [16], Digital Object Identifier (DOI) system [17], and TogoWS [18]. Although the Nature Publishing Group Linked Data Platform [19], the OpenCitations Project [20], and the Biotea Project [15] have already provided LOD that include citations, they have not provided citation contexts.

Our contribution is to provide LOD that include citation contexts in the life sciences domain. We provide three types of services that relate to the Colil database: a legacy web search interface (Colil), a SPARQL endpoint, and an ftp site to download dump files in RDF. The data needed for the Colil database have been extracted from open access papers deposited in the PubMed Central Open Access Subset (PMC-OAS) [16], which is made available under the Creative Commons License or similar licenses that generally provide users the rights to reuse and redistribute content. By using PMC-OAS, we can release the Colil database for reuse and redistribution under a Creative Commons License.

## Utility

### Search service description

**Fig. 1:**
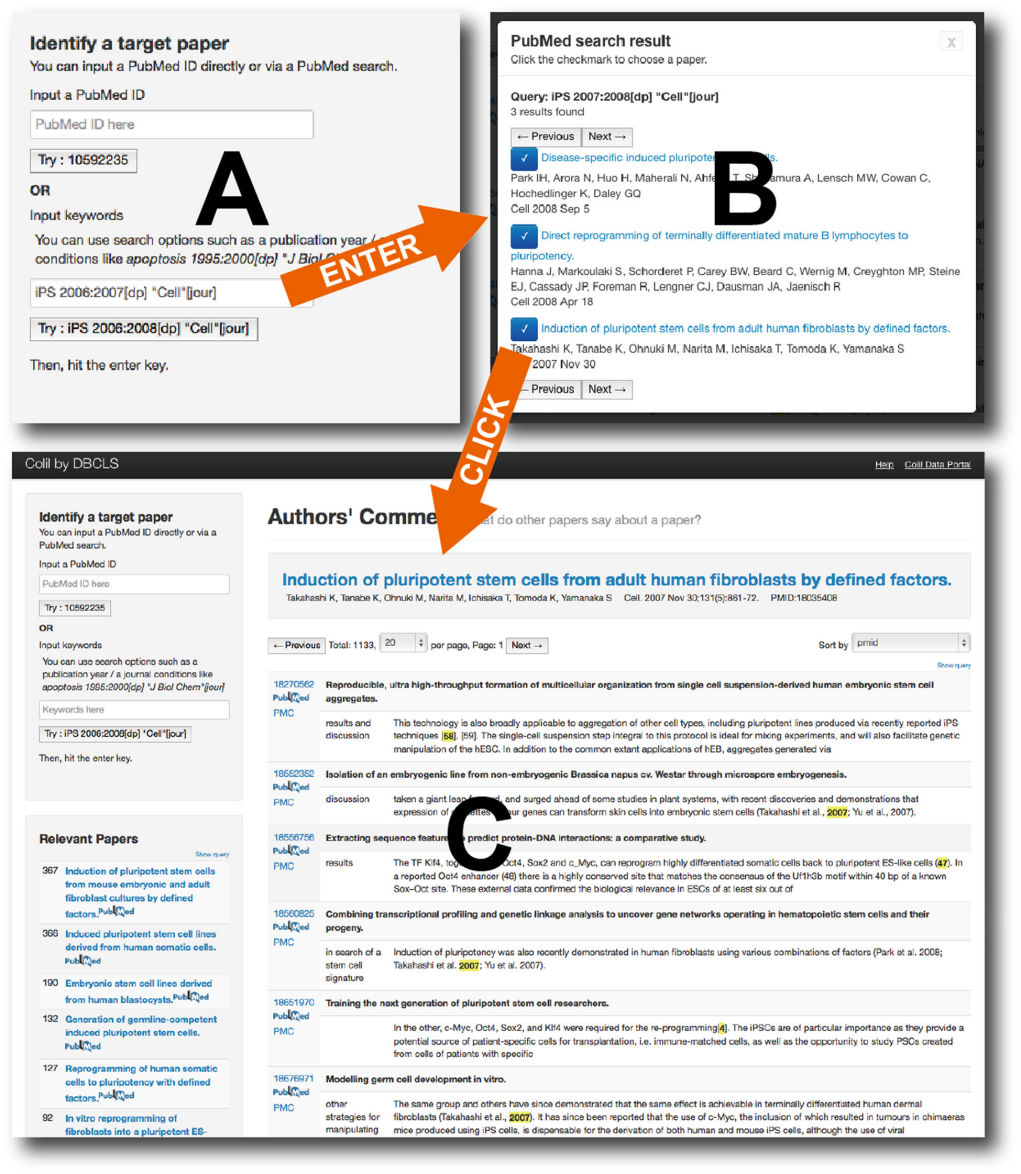
Colil search at a glance. **a** Searching for papers with keywords by using the PubMed search API. By typing ‘iPS 2007:2008[dp] “Cell” [jour]’ into the lower text box and hitting the enter key, the user can search for papers by using the PubMed search API and keywords. **b** Choosing a hit paper. Here, the user is provided with three hits in the modal dialog. By clicking the paper titled, “Induction of pluripotent stem cells from mouse embryonic and adult fibroblast cultures by defined factors.” Colil returns the corresponding results for the paper. **c** Displaying the search result. Here, the user is provided with the search result on the main page

**Fig. 2:**
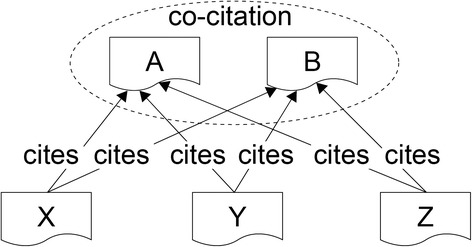
Co-citation. In the case where the papers (A, B) are co-cited by three different papers (X, Y, Z), the relevance score is 3

Colil searches for a cited paper in the Colil database and then returns a list of the citation contexts for it and its relevant papers based on co-citations. Users can choose a search method (*i.e.*, by PubMed ID or keywords). If a user initiates a search by typing a PubMed ID into the upper text box (Fig. 1a), Colil searches for a cited paper in the Colil database by this ID and then returns the corresponding citation contexts on the main page (Fig. 1c). If a user initiates a search by typing keywords into the lower text box (Fig. 1a), Colil searches for papers by using the PubMed search API [21]. Then, the search results are displayed in a modal dialog, where there is bibliographic information, including the title, authors, journal, year, volume, and issue (Fig. 1b). Each title is anchor text that links to the corresponding PubMed page. The user can choose one of the hit papers in the modal dialog, and then Colil returns the corresponding citation contexts on the main page (Fig. 1c). Colil’s main page (Fig. 1c) is divided into four areas, described below:

1. Search condition area (the upper left)
  In the search condition area, Colil accepts a user query with a PubMed ID or keywords. For keywords, users can use search options that are available in PubMed, such as publication year and journal name, as in ‘apoptosis 1995:2000[dp] “J Biol Chem” [jour]’.
2. Cited paper area (the upper right)
  In the cited paper area, Colil displays bibliographic information about the hit cited paper. This includes the title, authors, journal, year, volume, issue, and PubMed ID, and the title is anchor text that links to the corresponding PubMed page.
3. Citation context area (the lower right)
  In the citation context area, there is a list of the citation contexts for the hit cited paper. Colil highlights the reference markers, which are delimited by > > and < < in the citation contexts. Each row also includes the paper’s title and the section title of the citing paper. Furthermore, there are links to the corresponding pages at Colil, PubMed, and PMC. Rows in the list can be sorted in ascending or descending order for one of the following factors: PubMed ID, paper title, and section title. The list shows 20, 40, 60, or 100 results per page depending on the search condition. If the user clicks the anchor text for “Show query” on the upper side of the list, Colil shows the corresponding SPARQL query to obtain the result from our SPARQL endpoint.
4. Relevant paper area (the lower left)
  In the relevant paper area, there is a list of the relevant papers that are co-cited with the hit cited paper. Each row also includes a relevance score, the titles of relevant papers linking to the corresponding Colil page, and a link to the corresponding PubMed page. The relevance score is equal to the number of citing papers (Fig. 2). Rows in the list are sorted in descending order according to the relevance score, and only the top twenty relevant papers are displayed. If the user clicks the anchor text “Show query” on the upper side of the list, Colil shows the corresponding SPARQL query to obtain the result from our SPARQL endpoint.

### Example usage

A researcher wants to identify target genes of a microRNA (miRNA) by using databases that collect manually curated miRNA–gene interactions with an experimental support. For this purpose, the researcher needs to identify current trends and issues concerning the databases. To obtain this information, by using Colil, the researcher can search for papers relevant to the databases and utilize the citation contexts for the papers. The researcher initiates a search for papers by typing keywords “database miRNA target interactions manually curated experimental support”. As the result of the PubMed search (as of June 20th, 2015), Colil returns four papers, and three of them are original papers of widely-used databases such as TarBase, miRecords, and miRTarBase. Then, the researcher retrieves the corresponding citation contexts for the papers from Colil [see Additional file 1]; for example, the researcher can refer to the following citation contexts that help identify current trends and issues concerning the databases:

- Three databases are used to predict miRNA-target genes: TarBase (v6.0), miRecords (2013), and miRTarBase (2013), which host the largest collection of manually curated experimental data;
- The experimentally validated miRNA-target interactions information have been documented in various databases, such as TarBase, MiRecords, miRWalk, miRTarBase, and miRNAMAP;
- TarBase and miRTarBase document the experimentally-verified information on miRNA-target interactions along with their validation methods such as reporter genes, qPCR, western blotting, microarrays, proteomics, sequencing, and degradome sequencing data;
- For each miRNA, candidate targets have been inferred using data from six different databases (miRanda, PicTar, TargetScan, mirBase, miRTarget2, and TarBase) using the RmiR package from R Bioconductor (http://www.bioconductor.org/);
- Although miRTarBase has comprehensive information of miRNA targets from several organisms, it has scarce data on viral miRNA targets.

### Data retrieval

In addition to Colil, we also provide a SPARQL endpoint that makes it possible for those users who develop their own applications to retrieve data in the Colil database. In practice, Colil retrieves and uses data from the Colil SPARQL endpoint. For instance, Table 1 shows a SPARQL query to retrieve a set of citation contexts for a target paper. In addition, Table 2 shows a SPARQL query to retrieve a title list of relevant papers for a target paper; the list is ordered by the relevance score.

**Table 1:** SPARQL query to retrieve a set of citation contexts for a target paper

SPARQL query
PREFIX colil: <http://purl.jp/bio/10/colil/ontology/201303#>
PREFIX rdf: <http://www.w3.org/1999/02/22-rdf-syntax-ns#>
PREFIX rdfs: <http://www.w3.org/2000/01/rdf-schema#>
PREFIX togows: <http://togows.dbcls.jp/ontology/ncbi-pubmed#>
select ?Context where {
[] rdf:value ?Context ;
colil:mentions [
rdfs:seeAlso [
rdf:type colil:PubMed ;
togows:pmid “22135297”
]
].
}

**Table 2:** SPARQL query to retrieve a title list of relevant papers for a target paper; the list is ordered by the relevance score

SPARQL query
PREFIX colil: <http://purl.jp/bio/10/colil/ontology/201303#>
PREFIX rdf: <http://www.w3.org/1999/02/22-rdf-syntax-ns#>
PREFIX rdfs: <http://www.w3.org/2000/01/rdf-schema#>
PREFIX togows: <http://togows.dbcls.jp/ontology/ncbi-pubmed#>
select ?RelevantPaperTitle ?score where {
[] rdfs:seeAlso [
rdf:type colil:PubMed ;
togows:pmid “22135297”
];
colil:hasRelevantBibliographicResourceOf [
colil:RelevantScore ?score;
colil:hasRelevantPaperId [
rdfs:seeAlso [
rdf:type colil:PubMed ;
togows:ti ?RelevantPaperTitle
]
]
].
} order by desc(?score)

## Construction and content

### Construction

**Fig. 3:**
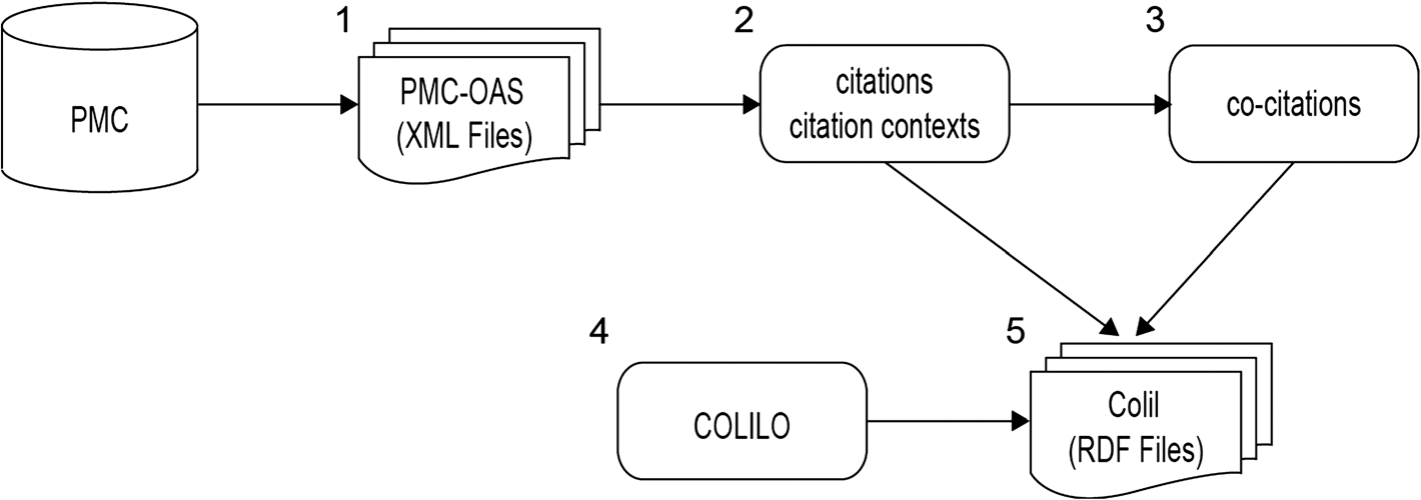
Process of building the Colil database. The full-text papers of PMC-OAS were obtained from PMC (1). Citations and citation contexts were identified from the papers (2), and co-citations were counted from citations (3). A newly compiled vocabulary called COLILO was developed (4), and the Colil database was built by using COLILO, citations, citation contexts, and co-citations (5)

Figure 3 illustrates the process that we followed to build the Colil database. We obtained full-text papers from PMC-OAS. Then, we extracted citations (references and reference markers) and citation contexts from these papers and counted co-citations from them. To build the Colil database, we used standard existing vocabularies, such as BIBO, DCTERMS, and DoCO, and compiled a new vocabulary. Below, we explain the construction method in more detail.

1. Obtaining full-text papers
  We downloaded PMC-OAS papers, which are downloadable as XML files from the NCBI (National Center for Biotechnology Information) FTP site at ftp://ftp.ncbi.nlm.nih.gov/pub/pmc. In March 2014, there were 713,029 papers. From these PMC-OAS papers, we then selected 670,497 papers that are indexed in PubMed; this was so that we could provide users with the PubMed search function to look up papers. Finally, of these 670,497 papers, we selected 545,147 that cited at least one PubMed-indexed paper.
2. Identifying citations and citation contexts
  We parsed the XML files to extract references and reference markers. These components are easily identifiable because the files contain XML tags such as ref and xref. We then matched each bibliographic information component to its corresponding reference marker(s). Different journals utilize different formats for reference markers, and some reference markers are grouped together within a pair of parentheses; these multiple reference markers are concatenated by a connector (*e.g.*, 1–6) or separated by a delimiter (*e.g.*, 1,2,3,4,6). We therefore built a parser for the different formats and types of reference markers. We used PubMed-indexed citations only.
  Next, we identified citation contexts located around the reference markers. A citation context can be multiple sentences, a sentence, or a fragment of a sentence. Based on our preliminary survey of citation contexts, we first defined the citation context as being the sentence containing the reference marker. Then, if the reference marker was located at the end of the sentence, we added the next sentence to the citation context. Finally, if the number of characters from the start of the citation context to the reference marker was over 240, only 240 characters before the reference marker and up to 240 characters after the reference marker were taken as the citation context.
3. Counting co-citations
  If a pair of papers is cited by at least two papers, we counted it as a co-citation. For co-citations, the more the pair is cited by other papers, the more they are deemed to be semantically related [22].
4. Developing a newly compiled vocabulary
  We used BIBO, DoCO, and DCTERMS as standard vocabularies to facilitate inter-operability and cross-resource exploration. These three standard vocabularies can be used to describe citations and bibliographic information, the component parts of a bibliographic document, and simple and generic resources, respectively. In addition, we compiled a new vocabulary called COLILO because the standard vocabularies were not comprehensive enough to express our data in RDF. Table 3 describes our new vocabulary consisting of six classes, four object properties, and three data type properties. To describe the vocabulary, we used the following font conventions: **classes**, ***object properties***, and ***data properties***. In addition, COLILO’s Base URI is http://purl.jp/bio/10/colil/ontology/201303#, and its prefix label is *colil*.

**Table 3:** New vocabularies

Classes	CitationPaper, Context, PubMed, ReferencePaper, RelevantBibliographicResource, RelevantPaper
Object properties	*CocitationWith*, *hasRelevantBibliographicResourceOf*, *hasRelevantPaperId*, *mentions*
Data properties	*Authors*, *pmcid*, *RelevantScore*
  The **ReferencePaper** class and the **CitationPaper** class describe the cited paper and the citing paper, respectively. The citation context is described by the **Context** class, and the ***mention*** property links the citation context to the cited paper. The **RelevantPaper** class is used to describe the co-cited paper (*i.e.*, relevant paper), and the ***cocitationWith*** property, which has characteristics of symmetry, links the cited paper to the relevant paper. The **RelevantBibliographicResource** is used to describe the co-citation, which is between co-cited papers and has a relevance score using the ***RelevantScore*** property. The ***hasRelevantBibliographicResourceOf*** property links the cited paper to the co-citation, and the ***hasRelevantPaperId*** property links the co-citation to the relevant paper. The **PubMed** class and the ***Authors*** property are used to describe the bibliographic metadata and its authors, respectively. The ***pmcid*** property is used to describe PMC IDs, which are identifiers of papers in PMC.
5. Building the Colil database
  We built the Colil database by using COLILO. Figure 4 shows an RDF graph that represents the relationship between the cited paper and the citing paper, which are represented as the **colil:ReferencePaper** and the **colil:CitationPaper** classes, respectively. We used the ***bibo:cites*** property to link the citing paper to the cited paper. We used the ***doco:contains*** property to represent a section in a document such as Introduction or Methods. Each section has a title and citation contexts, which are represented as the ***dcterms:title*** and the ***doco:contains*** properties, respectively. The citation context is represented as the **colil:Context** class, and the text is represented as the ***rdf:value*** property. We use the ***colil:mentions*** property to link the citation context to the cited paper.

**Fig. 4:**
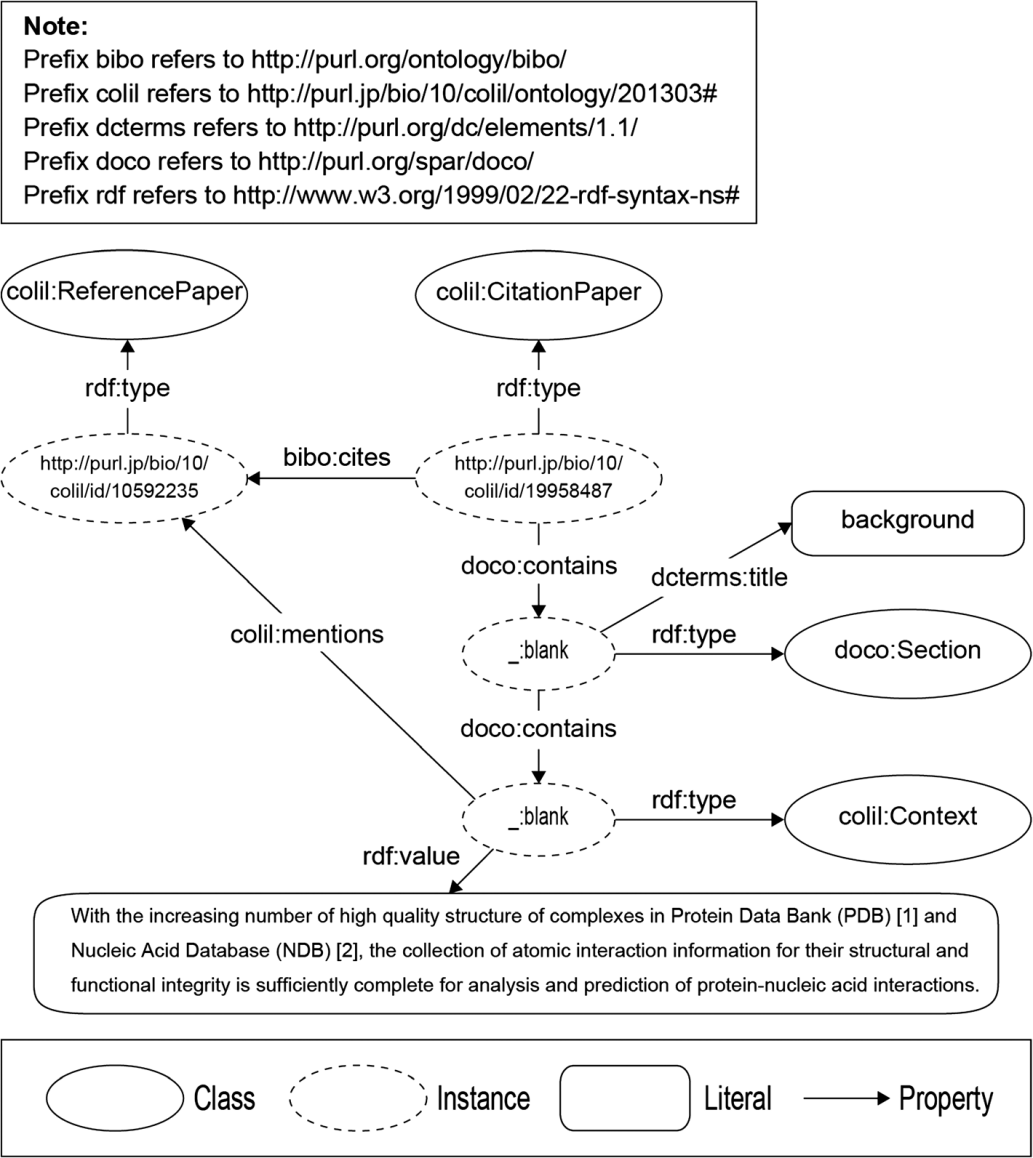
RDF graph representing the relationship between the cited paper and the citing paper
  Figure 5 shows an RDF graph that represents the relationship between the cited paper and a relevant paper. The **colil:RelevantPaper** class is used to represent the relevant paper, which is co-cited with another paper by other papers. We used the ***colil:cocitationWith*** property to link the cited paper to the relevant paper. To represent a relevance score between the cited paper and relevant paper, we provided the co-citation, which is represented as the **colil:RelevantBibliographicResource** class. The co-citation includes the relevance score, which is represented as the ***colil:RelevantScore*** property. The ***colil:hasRelevantBibliographicResourceOf*** property links the cited paper to the co-citation, and the ***colil:hasRelevantPaperId*** property links the co-citation to the relevant paper.

**Fig. 5:**
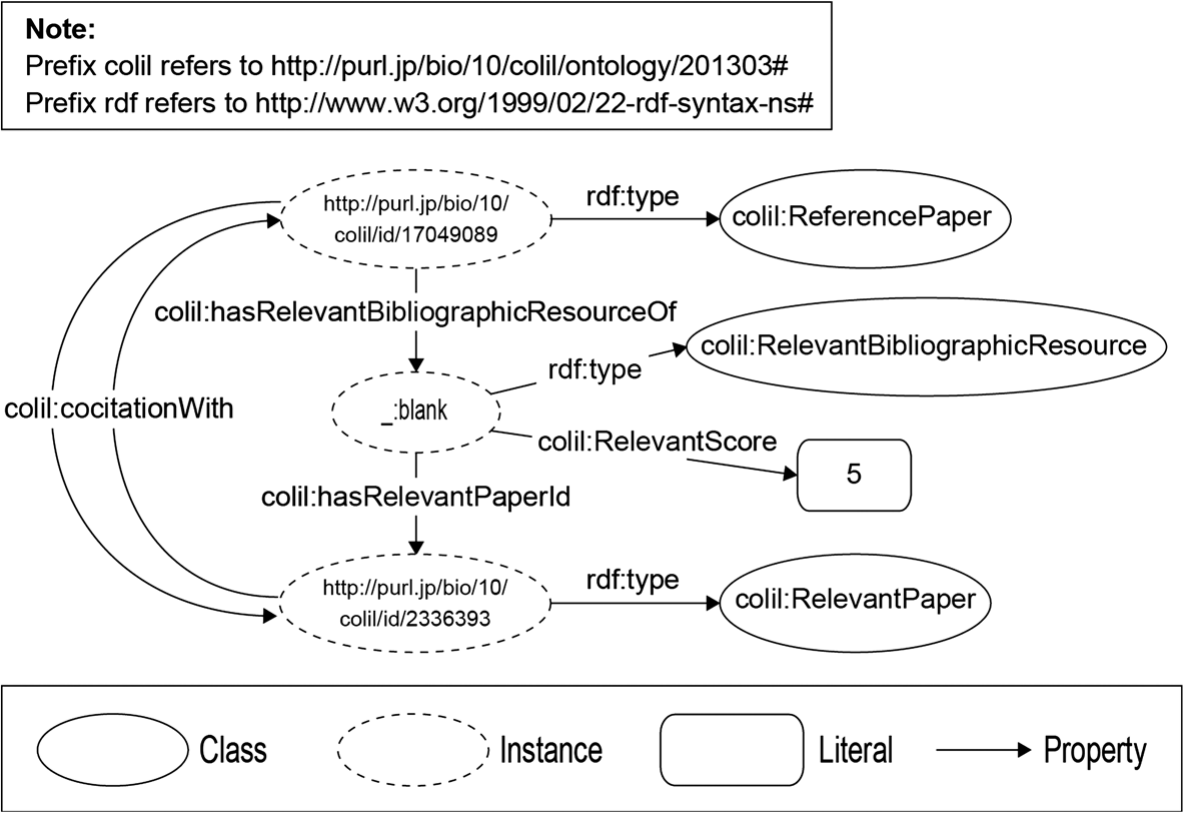
RDF graph representing the relationship between the cited paper and the relevant paper
  Figure 6 shows an RDF graph that represents the bibliographic metadata, links to the external resources, and the paper identifiers. We used PMC IDs and DOIs so that users could easily find the original paper. They are represented as the ***colil:pmcid*** and ***bibo:doi*** properties, respectively. The ***rdfs:seeAlso*** property is used to link external resources such as PMC, Biotea, DOI system, and TogoWS. Bibliographic metadata are represented as the **colil:PubMed** class by using the ***rdfs:seeAlso*** property. We used the ***togows:pmid***, ***colil:Authors***, ***togows:ti***, and ***togows:so*** properties to represent the bibliographic metadata of PubMed IDs, authors, title, and sources.

**Fig. 6:**
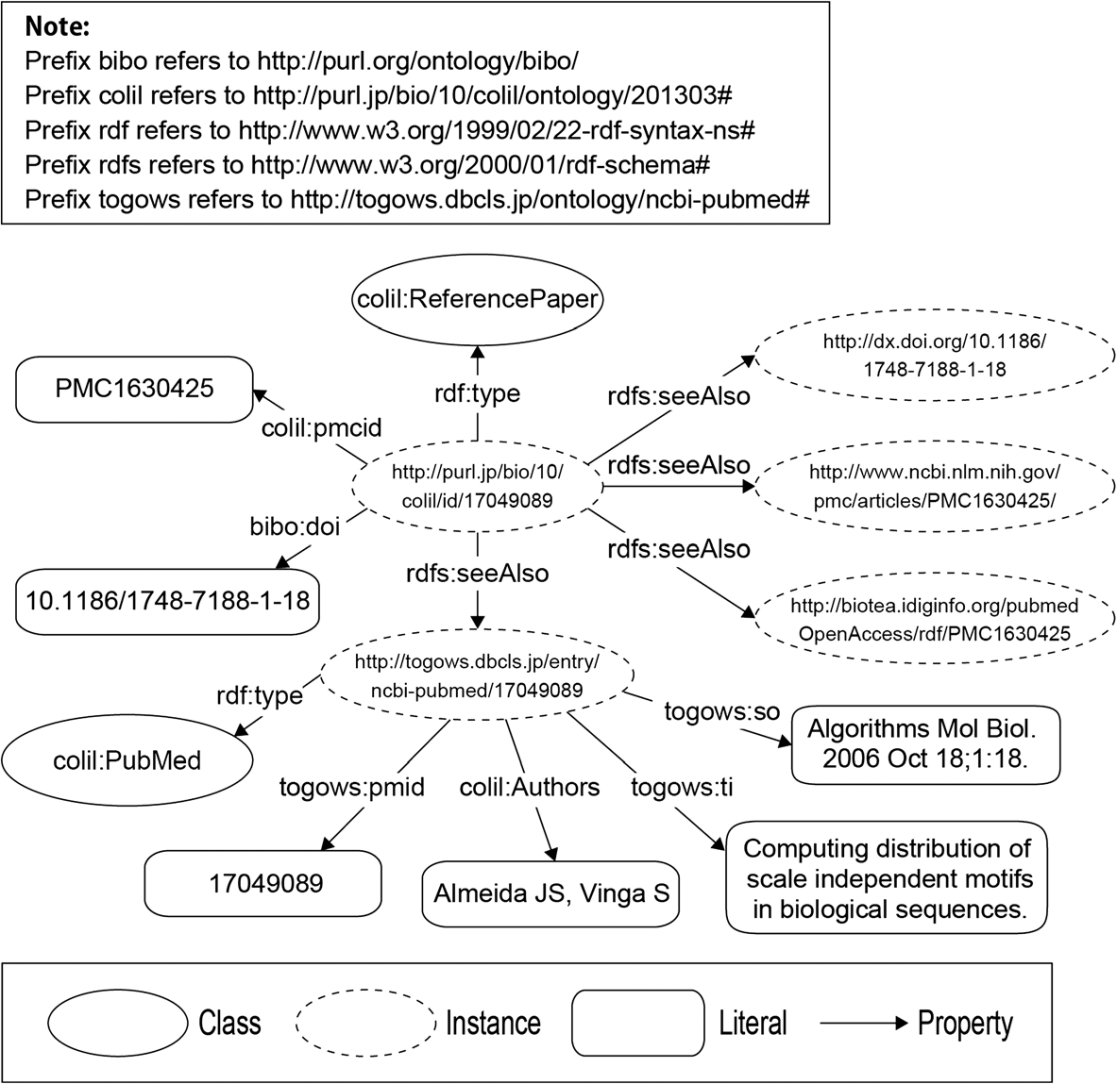
RDF graph representing the bibliographic metadata, links to external resources, and paper identifications

### Content

**Fig. 7:**
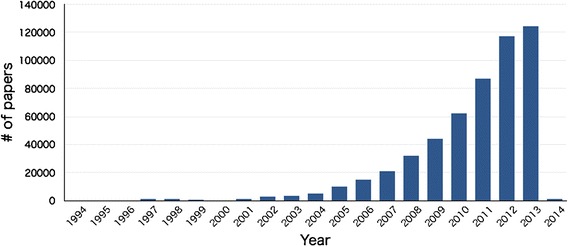
Growth of the number of PubMed-indexed papers published per year in PMC-OAS from 1994 to 2014

Over the past 10 years, the number of PubMed-indexed papers published each year in PMC-OAS has grown, with the most recent years showing an exponential growth (Fig. 7). We obtained 545,147 PubMed-indexed PMC-OAS papers that cited at least one PubMed-indexed paper; the obtained papers were distributed across 3,171 journals. The papers contained 24,684,765 citation contexts, and each of them cited an average of 41.5 PubMed-indexed papers. Table 4 lists the top 20 journals ranked according to the number of the PMC-OAS papers contained in each journal.

**Table 4:** Top 20 journals ranked according to the number of the PMC-OAS papers contained in each journal

Journal title	# of papers
PLOS ONE	82735
Nucleic Acids Research	10660
The Journal of Cell Biology	6001
BMC Public Health	5791
BMC Bioinformatics	5771
BMC Genomics	5736
Emerging Infectious Diseases	5534
The Journal of Experimental Medicine	4536
BMC Cancer	4074
PLOS Genetics	3929
International Journal of Molecular Sciences	3809
PLOS Pathogens	3572
Critical Care	3359
Scientific Reports	3299
Environmental Health Perspectives	3210
Evidence-based Complementary and Alternative Medicine: eCAM	2988
PLOS Computational Biology	2965
BMC Health Services Research	2731
Malaria Journal	2647
Journal of Medical Case Reports	2618

Conversely, 5,136,741 PubMed-indexed papers have been cited by at least one PMC-OAS paper; the cited papers correspond to approximately one-quarter of the entire PubMed entries and are distributed across 11,588 journals. Table 5 lists the top 20 journals ranked according to the number of cited papers contained in each journal.

**Table 5:** Top 20 journals ranked according to the number of cited papers contained in each journal

Journal title	# of papers
The Journal of Biological Chemistry	95169
Proceedings of the National Academy of Sciences of the United States of America	69118
PLOS ONE	38722
Journal of Immunology	31637
Nature	27947
Science (New York, N.Y.)	27760
Journal of Virology	26123
Biochemical and Biophysical Research Communications	25379
Cancer Research	24396
The Journal of Neuroscience	23357
Biochemistry	23153
Blood	22681
Journal of Bacteriology	21325
Nucleic Acids Research	20585
Biochimica et Biophysica Acta	20309
Lancet	20246
Brain Research	18750
Infection and Immunity	16983
Circulation	16898
The New England Journal of Medicine	16467

We found 27,832,062 co-citations in cited papers. To calculate the chances of a given paper having a co-cited paper in a different relevance score range, we classified 11 cases of co-citations according to their relevance scores (Table 6). Here, a chance is defined as the number of co-cited papers within the relevance score range divided by the total number of cited papers (5,136,741). For example, there are 2,126,896 co-cited papers with a relevance score of two and over, and the chance is approximately 41 %. Table 6 shows that this chance significantly decreases as the relevance score increases.

**Table 6:** The chances of a given paper having a co-cited paper in a different relevance score range

Relevance score range	Chance (%)
≥2	41.41
≥3	19.68
≥4	10.99
≥5	6.88
≥6	4.67
≥7	3.35
≥8	2.50
≥9	1.93
≥10	1.53
≥20	0.32
≥50	0.04

We released the Colil database, which consists of 445,671,312 triples, including approximately 6.8 million links to external resources. We use Virtuoso (VOS 7.1) [23] as the triple store; the Colil database is freely available for querying and browsing through the SPARQL endpoint at http://colil.dbcls.jp/sparql. There is a faceted web service interface for the Colil database at http://colil.dbcls.jp/fct. You can also access and search the Colil database with a free text query without registration. Complete dumps of the RDF data are downloadable through the FTP site at ftp://ftp.dbcls.jp/colil; the data are available for reuse and redistribution under a Creative Commons Attribution 2.1 Japan License. We also provide a portal site at http://colil.dbcls.jp/portal that includes an explanation of how to find the content and examples of querying the database by using the SPARQL endpoint.

## Discussion

### Support for writing citation contexts

As another use case, we envisage that Colil will help researchers to write citation contexts. In citation contexts, researchers must clarify the relationships between their paper and the papers cited within their paper [24]. However, this process may be difficult for some researchers, especially for non-English-speakers. In such cases, these researchers can learn how to write citation contexts more efficiently by observing the citation contexts that other researchers have made on a paper. We actually utilized Colil to write citation contexts in this paper and consider that Colil is suitable for this purpose. We continue to evaluate user experiences and will reflect the outcomes as Colil is developed further.

### Limitations of our approach

Due to the limited number of citing papers in the Colil database, there are cases where Colil is not able to provide enough citation contexts for a target paper. For example, papers cited in five or fewer citation contexts account for approximately 76 % of all the cited papers in the Colil database. One of the factors causing this issue is that the number of PubMed-indexed papers in PMC-OAS only corresponds to approximately 4 % of total PubMed entries at present; however, we expect that this situation will gradually improve because open access publications are gaining popularity and becoming the norm [9]. Figure 7 shows the exponential growth of PMC-OAS papers over recent years, and the number of the PubMed-indexed papers in PMC-OAS corresponds to approximately 14 % of the entire PubMed entries published from 2010 to 2014.

In contrast, there were 11,086 papers that were cited in 100 or more citation contexts. Although Colil may provide users with enough information and perspectives for these papers, it can be time consuming to find appropriate citation contexts for references. To alleviate this issue, we are considering providing users with a function to narrow down citation contexts according to their purposes. In previous studies, Amjad et al. [5] and Teufel et al. [25] proposed 6 and 12 categories for citation purposes, respectively, and showed that they could classify citation contexts into categories with good accuracy by using their proposed approaches. For future work, we will consider annotating citation contexts based on their work.

### Comparison of Colil with Microsoft Academic Search

As far as we know, Microsoft Academic Search (MAS) developed by Microsoft Research is the only search service to provide citation contexts in the life sciences domain except for Colil [26]. MAS is a freely accessible web-based search service and includes over 45 million records of academic publications. Although MAS has more indexes of the papers than does Colil, users cannot reuse and redistribute the data retrieved from it without previous permission. In addition, MAS has not been updated for about two years. On June 20th, 2015, we searched for the citation contexts at MAS and Colil for four papers retrieved with PubMed by using by the keywords “database miRNA target interactions manually curated experimental support” [Table 7]. MAS did not have the indexes of the papers that were published in or after 2013 since MAS was last updated in January 2013. The number of citation contexts for the papers is affected due to the lack of the citing papers that have been published in or after 2013. Colil was last updated on February 2nd, 2015 and has the indexes of four papers, and the number of citation contexts for each paper in Colil is higher than that in MAS. To keep up-to-date with the latest citing papers, we continue to update the Colil database twice a year.

**Table 7:** Comparison of search results for cited paper

PubMed ID	Year of publication	Cited paper (Hit or miss)		# of citation contexts	
		Colil	Microsoft academic search	Colil	Microsoft academic search
18996891	2009	Hit	Hit	220	42
21071411	2011	Hit	Hit	158	3
22135297	2012	Hit	Miss	88	N/A
23376192	2014	Hit	Miss	1	N/A

### Problem of identifying citation contexts

A citation context can be defined as sentences that comment on the work of a citing paper. Based on our preliminary survey of citation contexts, we defined a citation context as a sentence that contains a reference marker, except when the reference marker is located at the end of the sentence. We call a sentence that contains a reference marker an “explicit citing sentence” [5]. In this regard, some studies proposed methods to classify sentences as citation contexts from ones appearing around an explicit citing sentence [5]. Qazvinian and Radev [27] showed experimentally that they could retrieve important information to survey scientific papers from sentences appearing around explicit citing sentences. To provide more comprehensive information, we are planning to improve our method of identifying a citation context based on their studies.

## Conclusions

The Colil database includes citations, citation contexts, and co-citations extracted from full-text papers of PMC-OAS. To help users to utilize the database in their respective research environments, we provide three types of services: a Colil search service, a SPARQL endpoint, and an ftp site. Colil can facilitate finding a set of citation contexts and relevant papers based on co-citations for a target paper. This should help researchers to comprehend life sciences papers in a research area more efficiently and make their biological research more efficient..

## Availability and requirements

The Colil database is available under a Creative Commons Attribution 2.1 Japan License and can be downloaded at ftp://ftp.dbcls.jp/colil. COLILO is freely available under a Creative Commons Zero License at http://purl.jp/bio/10/colil/ontology/201303. Colil is available for public access at http://colil.dbcls.jp/browse/papers/.

## Additional file

Additional file 1:
**Retrieving sets of citation contexts for papers with PubMed ID 18996891, 21071411, 22135297, and 23376192.** (XLSX 66 kb)

## Abbreviations

BIBO: Bibliographic Ontology
Colil: Comments on Literature in Literature
COLILO: Comments on Literature in Literature Ontology
DCTERMS: Dublin Core Metadata Initiative Metadata Terms
DoCO: Document Components Ontology
DOI: Digital Object Identifier
LOD: Linked Open Data
MAS: Microsoft Academic Search
miRNA: microRNA
PMC: PubMed Central
PMC-OAS: PubMed Central Open Access Subset
RDF: Resource Description Framework
